# Application of Recombinase-Based In Vivo Expression Technology to *Bifidobacterium longum* subsp. *longum* for Identification of Genes Induced in the Gastrointestinal Tract of Mice

**DOI:** 10.3390/microorganisms8030410

**Published:** 2020-03-13

**Authors:** Hiroka Koguchi, Natsumi Ishigami, Mikiyasu Sakanaka, Kako Yoshida, Sayaka Hiratou, Mina Shimada, Satoru Fukiya, Kei Sonoyama, Atsushi Yokota

**Affiliations:** 1 Laboratory of Microbial Physiology, Research Faculty of Agriculture, Hokkaido University, Sapporo 060-8589, Japan; hirosa@dtu.dk (H.K.); miksak@dtu.dk (M.S.); yokota@chem.agr.hokudai.ac.jp (A.Y.); 2 Laboratory of Food Biochemistry, Research Faculty of Agriculture, Hokkaido University, Sapporo 060-8589, Japan; ksnym@chem.agr.hokudai.ac.jp

**Keywords:** bifidobacteria, *Bifidobacterium longum* subsp. *longum*, R-IVET, in vivo gene expression, Cre recombinase, gut microbiota, qRT-PCR

## Abstract

Bifidobacteria are one of the major components in human gut microbiota and well-known as beneficial microbes. However, clarification of commensal mechanisms of bifidobacteria in the intestines is still ongoing, especially in the presence of the gut microbiota. Here, we applied recombinase-based in vivo expression technology (R-IVET) using the bacteriophage P1 Cre/*loxP* system to *Bifidobacterium longum* subsp. *longum* 105-A (*B. longum* 105-A) to identify genes that are specifically expressed in the gastrointestinal tract of conventionally raised mice. Oral administration of the genomic DNA library of *B. longum* 105-A to conventionally raised mice resulted in the identification of 73 in vivo-induced genes. Four out of seven tested genes were verified in vivo-specific induction at least in the cecum by quantitative reverse transcription PCR. Although there is still room for improvement of the system, our findings can contribute to expanding our understanding of the commensal behavior of *B. longum* in the gut ecosystem.

## 1. Introduction

Bifidobacteria are one of the major components in the gut microbiota of humans, especially infants [1,2,3]. Currently, more than 10 species of *Bifidobacterium* are known to colonize the human gut [4]. However, the occurrence of each in the gut differs depending on the species [5]. *Bifidobacterium longum* subsp. *longum* (*B. longum*) is one of the most representative human gut-associated bifidobacteria. A recent study has reported that *B. longum* is the most ubiquitously and highly distributed among bifidobacteria across the human lifespan [6]. *B. longum* is also prevalent across various mammalian species [7]. Certain strains of *B. longum* are reported to provide hosts with health benefits [8,9]. Considering such ecological roles of *B. longum*, clarification of why and how they colonize the gut and interact with the host is important. The importance of several proteins (enzymes) in terms of bifidobacterial gut colonization and/or host physiology has been reported in *B. longum* so far. Several glycosidases and transporters are involved in the proliferation of *B. longum* in the gut through assimilation of host glycans and dietary fibers [10,11]. The cell surface fimbrial protein binds to host colonic mucin to possibly enhance the colonization ability in the gut [12]. A serine protease inhibitor produced by *B. longum* possesses immune-modulating properties in the host [13]. Nevertheless, in vivo commensal mechanisms of *B. longum*, especially in the presence of the gut microbiota, are still unexplored, probably because of the limitation of comprehensive information on in vivo gene expression [14,15]. Even for other *Bifidobacterium* species, in vivo transcriptome analyses, such as DNA microarray and RNA-sequencing, are limited [16,17,18]. 

**Figure 1:**
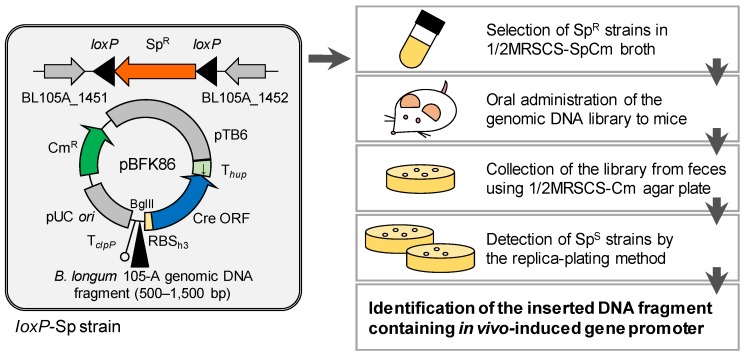
Overview of the recombinase-based in vivo expression technology (R-IVET) system constructed in this study. The *loxP*-Sp strain harbored a *loxP*-Sp^R^-*loxP* cassette that was inserted between BL105A_1451 and BL105A_1452 on the chromosome of *B. longum* 105-A. Random DNA fragments of *B. longum* 105-A were independently inserted upstream of the promoterless Cre gene in pBFK86. The resulting plasmids were introduced into the *loxP*-Sp strain, generating the genomic DNA library consisting of ~120,000 clones. The library was cultured in Sp-containing medium to exclude Sp^S^ strains in which the Cre gene was expressed by the DNA fragment with in vitro promoter activity. The library was then administered orally to mice and collected from feces. Finally, the Sp^S^ strains in which the Cre gene had been expressed during passage through the gastrointestinal tract were identified to determine in vivo-induced gene promoters. Sp^R^, spectinomycin resistance; Sp^S^, spectinomycin sensitive; Cm^R^, chloramphenicol resistance.

An effective approach to resolve this issue is to use recombinase-based in vivo expression technology (R-IVET) that enables identification of bacterial genes expressed specifically in vivo or in specific environmental conditions [19,20,21,22,23,24]. Basic R-IVET applies the Cre/*loxP* site-specific recombination system from bacteriophage P1 (Figure 1) [19]. In R-IVET, an antibiotic resistance gene that is sandwiched by two *loxP* sites is inserted into the chromosome of the host strain. A promoterless Cre gene located downstream of a random DNA fragment from the host genome is provided by a plasmid. Promoter activity of the DNA fragment induces the Cre expression and the site-specific recombination between two *loxP* sites results in exclusion of the antibiotic resistance gene from the chromosome. Consequently, based on evaluation of the antibiotic susceptibility of strains, in vivo-induced genes can be identified. One of the characteristics for R-IVET is that in vivo expression can be evaluated in each single cell by the irreversible recombination reaction. Therefore, this technology is advantageous to detect in vivo-induced genes, including transiently and locally expressed genes, even in low persistent bacterial strains in certain environments. The data obtained by R-IVET can provide valuable information to understand in vivo bacterial behavior, especially when integrated with other types of transcriptomic data such as DNA microarray and RNA-sequencing.

Here, we applied the R-IVET system to *B. longum* 105-A to identify genes that are specifically expressed in vivo. Oral administration of the genomic DNA library of *B. longum* 105-A to conventionally raised mice resulted in identification of 73 genes induced in the gastrointestinal tract. Quantitative reverse-transcription PCR (qRT-PCR) analysis verified the in vivo-induced expression of four out of seven tested genes in the cecum of the mice. These findings can contribute to advance our understanding of commensal mechanisms of *B. longum* in the gut ecosystem.

## 2. Materials and Methods 

### 2.1. Bacterial Strains and Culture Conditions

The representative bacterial strains used in this study are listed in Table 1. The *Escherichia coli* DH5α strain was used as a DNA cloning host and grown aerobically in Luria-Bertani (LB) medium. *B. longum* 105-A (JCM 31944; RIKEN BioResource Research Center [25]) was anaerobically grown at 37 °C in a half concentration of de Man, Rogosa, and Sharpe (MRS) medium [26] supplemented with 0.34% (*w*/*v*) sodium ascorbate and 0.02% (*w*/*v*) cysteine-HCl (1/2MRSCS medium). Anaerobic cultivation was carried out in an anaerobic chamber (80% N_2_, 10% CO_2_, and 10% H_2_; Coy Laboratory Products, Inc., Grass Lake, MI, USA) or a closed pouch with an AnaeroPack (Mitsubishi Gas Chemical, Tokyo, Japan). When necessary, antibiotics were added to the media as follows: spectinomycin (Sp; 75 μg/mL) and chloramphenicol (Cm; 10 μg/mL for *E. coli* and 2.5 μg/mL for *B. longum*), if not indicated.

### 2.2. Animal Experiments

Animal experiments were approved by the Animal Use Committee of Hokkaido University (no. 17-0050, approved on 15 March 2018). Animals were maintained following the Hokkaido University guidelines for the care and use of laboratory animals. Five-week-old female BALB/c mice were purchased from Japan SLC (Shizuoka, Japan). Mice were housed in standard plastic cages in a temperature-controlled environment (23 ± 2 °C) with a 12-h light/dark cycle and allowed free access to water and food. For the first and second R-IVET experiments, mice (*n* = 4 per each experiment) were acclimatized for 1 week by feeding on a standard chow diet (MR stock; Nosan Corporation, Yokohama, Japan). After administration of the R-IVET library, the mice continuously fed on the same diet. 

For the third and fourth R-IVET experiments, an AIN-93G control diet (Supplementary Table S1) was fed to the mice (*n* = 2 per each experiment) for 2 weeks before the administration. On the day of R-IVET library administration, the mice were fed the AIN-93G-based diet containing 6% (*w*/*w*) 1-kestose (kindly provided by B Food Science Co., Ltd., Tokyo, Japan) at the expense of maltodextrin to increase the persistence of *B. longum* 105-A in the mouse intestines. After the administration, the mice continuously fed on the same diet. BALB/c mice (*n* = 6) were also acclimatized and reared in the same manner for qRT-PCR analysis of the identified genes in the screening described in Section 2.7.

### 2.3. Generation of the B. longum Strain for the R-IVET System

The Sp resistance (Sp^R^) gene flanked by two *loxP* sites (*loxP*-Sp^R^-*loxP*) was inserted between BL105A_1451 (putative aminotransferase) and BL105A_1452 (galactoside transport protein) on the chromosome of *B. longum* 105-A by double-crossover recombination as described previously [27]. Sanger sequencing was carried out to confirm that mutation other than those expected had not occurred. The resulting mutant was designated as the *loxP*-Sp strain.

The plasmid for integration of *loxP*-Sp^R^-*loxP* was constructed as described below. The Sp^R^ gene was amplified by PCR from pBS423 [27] and inserted into the SwaI site (between two *loxP* sequences) of pULwL [28], which yielded pBFH23. Then, two homologous DNA regions to the BL105A_1451 locus (designated HR1) or BL105A_1452 locus (HR2) were amplified by PCR from *B. longum* 105-A genomic DNA and inserted into the EcoRI and BamHI sites of pBFH23, respectively. The resulting plasmid, pBFH35, was used as a DNA template for PCR amplification of a DNA fragment containing HR1, *loxP*-Sp^R^-*loxP*, and HR2 in this order. The fragment was inserted into pBFS423Δ*repA* [27] lacking the Sp^R^ gene, which was amplified by inverse PCR. The primers and DNA cloning strategies are listed in Table 2 nos. 1–5.

### 2.4. Construction of a Plasmid Harboring the Cre Gene for the R-IVET System

#### 2.4.1. Cloning of a Promoterless Cre Gene with an RBS 

A Cm resistance gene was amplified by PCR from pBFS38 [29] and cloned into the ScaI- and NsiI-digested pKKT427 [30] fragment containing the pTB6 replicon and pUC *ori*, resulting in the *E. coli*-*Bifidobacterium* shuttle vector pBFS63. Then, promoterless Cre genes with different ribosome-binding sites (RBSs) (RBS_h4_, 5′-GAAGGATGCT-3′; RBS_h3_, 5′-GAAGGATGC-3′) [31] were amplified by PCR from bacteriophage P1 genomic DNA [32] and inserted into the BglII site of pBFS63, generating pBFH65-5 and pBFK71, respectively. pBFH65-4 with a spontaneous RBS mutation (5′-GAGGATGCT-3′, hereafter designated RBS_h4′_) was also obtained incidentally. The primers and DNA cloning strategies are indicated in Table 2 nos. 6–8. 

#### 2.4.2. Insertion of a Transcriptional Terminator 

The following four transcriptional terminators were used for the analysis: T*_las_* (a terminator for the latic acid synthesis operon of *Lactococcus lactis* [33]); T*_rps9_* (putative terminator for the 30S ribosomal protein S9 gene of *B. longum* 105-A); T*_leuB_* (terminator for the 3-isopropylmalate dehydrogenase gene of *Corynebacterium glutamicum* ATCC 13032 [34]); T*_clpP_* (modified terminator for the *clpP* operon of *Bifidobacterium breve* UCC2003 [35]). T*_las_*, T*_rps9_*, and T*_leuB_* were amplified by PCR and inserted into the BglII site of pBFK71 (upstream of the promoterless Cre gene), yielding pBFH78, pBFH80, and pBFK85, respectively. pBFK86, in which T*_clpP_* is inserted upstream of the promoterless Cre gene, was constructed as follows. The T*_clpP_* stem-loop, BglII site, RBS_h3_, and Cre ORF were amplified by PCR in this order and inserted into the BglII site of pBFS63 using an In-Fusion^®^ HD cloning kit (Clontech Laboratories, Inc., Mountain View, CA, USA). The 5′-protruding end of BglII-digested pBFS63 was removed by mung bean nuclease (New England Biolabs, Inc., Ipswich, MA, USA). The primers, DNA templates, and cloning strategies are listed in Table 2 nos. 9–12.

#### 2.4.3. Insertion of a Promoter

P*_cscBA_*, the promoter of a putative operon including sucrose permease and β-fructofuranosidase genes [29], was amplified by PCR from *B. longum* 105-A genomic DNA and inserted into the BglII site (upstream of the promoterless Cre gene) of pBFK86, resulting in pBFK94. The primers and cloning methods are indicated in Table 2 no. 13.

### 2.5. Evaluation of Basal Cre Expression Levels in Promoterless Cre Plasmids

Basal expression levels of Cre from plasmids harboring the promoterless Cre gene were evaluated by measuring the retention ability of the Sp^R^ gene in the *loxP*-Sp strain. A plasmid lacking the Cre gene (pBFS63) was used as a negative control. Each plasmid was introduced into the *loxP*-Sp strain by electrotransformation as described previously [27]. After electroporation of the plasmid, the cells were anaerobically incubated in 1/2MRSCS-Sp broth for 3 h and then spread on 1/2MRSCS-Cm agar plates. Two colonies of the transformants were picked up and independently transferred to 1/2MRSCS-Cm broth. After overnight incubation, the cultures were spread on 1/2MRSCS-Cm agar plates and the obtained colonies were replicated on 1/2MRSCS-Cm agar plates with or without Sp. The proportion of Sp^R^ strains was determined by dividing the number of Sp^R^ strains by that of the tested strains. Retention of the Sp^R^ gene was confirmed by colony PCR using the primer pair Pr-Blo0099/Pr-Blo0100 (Table 2 no. 14). 

The *loxP*-Sp strain harboring pBFK94 (Cre expression plasmid under the control of P*_cscBA_*) was also used for the evaluation as follows. Competent cells of the *loxP*-Sp strain were electroporated with pBFK94, resuspended in 1/2MRSCS-Sp broth, and then incubated for 3 h. The culture was further incubated on 1/2MRSCS-Cm agar plates containing 1% (*w*/*v*) glucose (uninduced condition) or 1% raffinose (induced condition) as a sole carbohydrate source. The proportion of Sp^R^ strains was determined as described above.

### 2.6. Construction of the Genomic DNA Library

A plasmid library was constructed by inserting a bifidobacterial genomic DNA fragment into the BglII site of pBFK86 (Figure 1). Genomic DNA (480 μg) from *B. longum* 105-A was partially digested with Sau3AI, and then 500–1500-bp DNA fragments were collected using a MinElute gel extraction kit (Qiagen, Hilden, Germany) after agarose gel electrophoresis. The DNA fragments were ligated into BglII-digested pBFK86, and the ligation products were cloned in *E. coli* DH5α. Plasmids were extracted from ~80,000 *E. coli* transformants and introduced into the *loxP*-Sp strain by electrotransformation. After electroporation, the cells were anaerobically incubated for 1 h in 1/2MRSCS-Sp broth and then spread on 1/2MRSCS-Cm agar plates. After 2 days of incubation, ~120,000 *Bifidobacterium* transformants were anaerobically suspended in 1/2MRSCS broth supplemented with 10% glycerol and the resulting R-IVET library was dispensed into aliquots and frozen at −80 °C until use. The library suspension was also incubated on 1/2MRSCS-Cm and 1/2MRSCS-SpCm agar plates to determine the proportion of Sp^R^ strains in the library. Colony PCR was carried out using a primer pair Pr-Blo0277/Pr-Blo0318 (Table 2 no. 15), and the resulting PCR products were used for Sanger sequencing to determine the size and nucleotide sequence of the DNA fragments inserted upstream of the Cre gene.

### 2.7. Screening for In Vivo-Induced Genes in B. longum

#### 2.7.1. First and Second Trials

An overview of the screening is shown in Figure 1. The R-IVET library was thawed on ice, and then ~10^7^ cells (100 µL) were inoculated into 5 mL 1/2MRSCS-SpCm broth and cultured anaerobically overnight. The culture was transferred to 40 mL fresh 1/2MRSCS-SpCm to an initial OD_660_ of 0.05 and cultured anaerobically to OD_660_ of 0.8–1.0. The cells (2 mL culture) were washed once with anaerobically stored phosphate-buffered saline (PBS) and then resuspended in 200 µL of the same buffer. The inoculum (approximately 10^9^ cells) was then administered orally to a 6-week-old mouse (*n* = 4 for first and second trials, respectively) that was fed with MR stock. At 3 and 12 h after oral administration, fresh feces were collected from the mice, homogenized with PBS, and plated on a 1/2MRSCS agar plate supplemented with Cm (10 μg/mL). After 60 h of incubation, the colonies had replicated on 1/2MRSCS-Cm and 1/2MRSCS-SpCm agar plates. Subsequently, Sp-sensitive (Sp^S^) clones were used for colony PCR and Sanger sequencing. The colony PCR was conducted using the primer pair Pr-Blo0099/Pr-Blo0100 (Table 2 no. 14) to confirm excision of the Sp^R^ gene, whereas Sanger sequencing following colony PCR using a primer pair Pr-Blo0277/Pr-Blo0318 (Table 2 no. 15) was carried out to determine the nucleotide sequence of the DNA fragments inserted upstream of the Cre gene. The complete genome sequence of *B. longum* 105-A (GenBank accession no. AP014658.1) [36] was used as a reference. Consequently, inserted DNA fragments containing an intergenic region(s) were identified as in vivo-induced promoters. Genes located downstream of the identified promoters were subjected to blastp analysis (BLAST + v2.2.25) against the database of Clusters of Orthologous Groups (COG) [37] and assigned to COG categories. 

#### 2.7.2. Third and Fourth Trials

The third and fourth trials of the screening were conducted as described in Section 2.7.1 except for differences in the animal rearing conditions and timing for collection of fecal samples after administration of the R-IVET library. The inoculum (approximately 10^9^ cells) of the R-IVET library was prepared as described above and orally administered to 7-week-old mice (*n* = 2 for third and fourth trials, respectively) that were fed the AIN-93G control diet. After the administration, the diet was changed to the AIN-93G-based, 1-kestose containing diet, enabling *B. longum* 105-A to colonize the mouse intestines (see Section 2.2). Fresh feces were collected from two mice in each trial at 4 days after the administration and used for screening in vivo-induced genes as described in Section 2.7.1. 

### 2.8. RNA Extraction and qRT-PCR Analysis to Verify Specific In Vivo Gene Expression

#### 2.8.1. Administration of *B. longum* 105-A Harboring pBFS63

**Figure 2:**
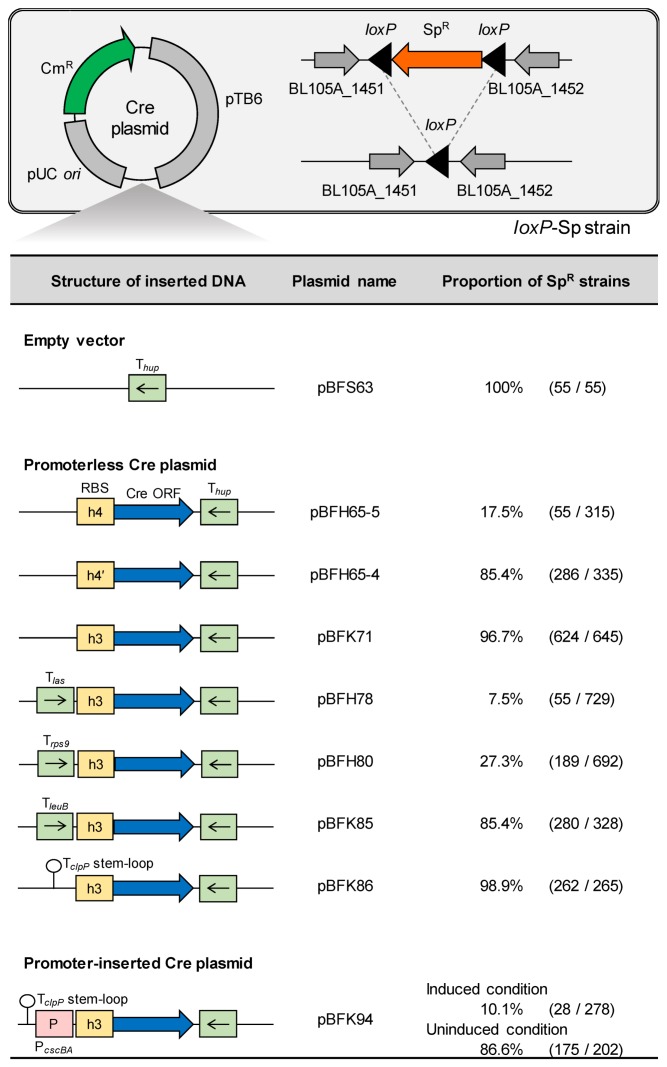
Proportion of Sp^R^ strains when each Cre expression plasmid was independently introduced into the *loxP*-Sp strain. Detailed methods are described in Section 2.5 of the Materials and Methods. The proportion of Sp^R^ strains in the tested strains is shown as a percentage. The values in parenthesis indicate the number of Sp^R^ strains among the tested strains. Sp^R^, spectinomycin resistance; Cm^R^, chloramphenicol resistance. Green box with an arrow, terminator; yellow box, ribosome-binding site (RBS); pink box, promoter.

BALB/c mice (*n* = 6), which were acclimatized and reared with the AIN-93G control diet, were used for this experiment. *B. longum* 105-A harboring pBFS63 (Figure 2) was cultured in 1/2MRSCS-Cm broth, and the cells were collected for administration as described in Section 2.7.1. Inoculum containing approximately 10^9^ cells was administrated orally to the mice once per day for 3 days to ensure a population of *B. longum* 105-A cells in the cecal microbiota. After the first administration, the diet was changed to the AIN-93G-based diet containing 6% (*w*/*w*) 1-kestose until dissection. At 2 days after the third administration, the mice were anesthetized using sevoflurane (Wako Pure Chemicals, Osaka, Japan) and sacrificed by exsanguination of the carotid artery. The cecum was excised after a laparotomy. Then, 68–194 mg cecal contents were collected and resuspended in five volumes of RNAprotect bacteria reagent (Qiagen). The resuspended cecal contents were pretreated following the manufacturer’s instructions, immediately frozen in liquid nitrogen, and then stored at −80 °C until use.

#### 2.8.2. RNA Extraction and qRT-PCR Analysis

*B. longum* 105-A harboring pBFS63 was cultured anaerobically in 1/2MRSCS-Cm broth until the mid-log phase (OD_660_ = 0.5–0.7). The cells were pretreated with RNAprotect bacteria reagent (Qiagen) and total RNA was extracted as described in our previous study using enzymatic cell wall digestion and mechanical cell disruption with zirconia beads [38]. Total RNA was also extracted from pretreated mouse cecal contents (see Section 2.8.1) using the same protocol. cDNA synthesis and qRT-PCR analysis were conducted as described previously [38]. A relative standard curve method was used to calculate the relative expression of the target gene against the reference gene. Data obtained from the *B. longum* 105-A in vitro culture (*n* = 4) and the same strain-administrated mice (*n* = 6) were statistically compared by Student’s or Welch’s two-tailed *t*-tests after testing the equality of variance by the *F*-test. *p*-values of less than 0.05 were considered as significantly different. 

Candidate genes identified by the R-IVET analysis were subjected to qRT-PCR analysis to verify in vivo-specific expression: BL105A_0130 (presumable pilin subunit for Tad-pili), BL105A_0467 (putative adhesin), BL105A_0547 (ATPase of the ABC transporter), BL105A_1291 (serine protease inhibitor), BL105A_1293 (galactoside transport protein), BL105A_1294 (glycoside hydrolase family 32 β-fructofuranosidase), BL105A_1798 (putative glycosyltransferase), and BL105A_1894 (raffinose transport system permease protein). Among them, BL105A_1294 was not identified in the R-IVET analysis, but this gene was used as an expected positive control gene for in vivo-specific expression because the β-fructofuranosidase encoded by this gene is necessary to degrade 1-kestose in the mouse diet [39]. BL105A_1946 (*rnpA* encoding the RNase P protein component) was used as a reference gene [17,38]. Gene-specific primers for qRT-PCR analysis are shown in Table 2 nos. 16–24. The inserted fragments of the R-IVET clones corresponding to the genes subjected to qRT-PCR analysis were subjected to promoter prediction. First, genomic positions of the inserted fragments were verified in the *B. longum* 105-A genome. Subsequently, bacterial vegetative promoters were predicted in the inserted fragments using Genetyx ver.12 (GENETYX corp., Tokyo, Japan) with the consensus sequence (5′-TATAAT-3′ as the −10 region and 5′-TTGACA-3′ for the −35 region). 

## 3. Results

### 3.1. Development of the R-IVET System for B. longum 105-A 

We generated the *loxP*-Sp strain in which *loxP*-Sp^R^-*loxP* was inserted into the intergenic region between BL105A_1451 and BL105A_1452 on the chromosome of *B. longum* 105-A (Figure 1). When grown in 1/2MRSCS liquid medium, the growth abilities were indistinguishable between *loxP*-Sp and wild-type strains (generation time: 73.2 min for former and 72.8 min for latter (an average of biological duplicates)). After 41 generations of culture without Sp, all 307 clones of the *loxP*-Sp strain retained the phenotype of Sp^R^. Colony PCR also showed that all 36 tested clones harbored the Sp^R^ gene. These results indicated that the Sp^R^ gene was stably maintained in the chromosome of *B. longum* 105-A without exerting negative effects on their in vitro growth. 

Next, we constructed a promoterless Cre plasmid suitable for the R-IVET system. The R-IVET system evaluates the promoter activity of a DNA fragment inserted upstream of the promoterless Cre gene based on phenotypic examination of the Sp^R^ ability. Therefore, basal Cre expression should be suppressed in the absence of the inserted DNA fragment. First, to determine a suitable RBS, three plasmids, pBFH65-5, pBFH65-4, and pBFK71, carrying promoterless Cre genes with different RBSs were used for the evaluation (Figure 2). When these plasmids were independently introduced into the *loxP*-Sp strain, 96.7% of pBFK71-carrying transformants showed the Sp^R^ phenotype. This result was comparable with that of transformants of pBFS63 lacking promoterless Cre genes (Figure 2). In contrast, the pBFH65-5- or pBFH65-4-carrying transformants retained the Sp^R^ phenotype at only 17.5% and 85.4%, respectively. These results indicated that pBFK71 strongly suppressed basal expression of the promoterless Cre gene (Figure 2).

Subsequently, transcriptional terminators were inserted upstream of the promoterless Cre gene in pBFK71 to construct the plasmids pBFH78, pBFH80, pBFK85, and pBFK86 to further suppress the basal Cre expression. Evaluation was then conducted as described above (Figure 2). The insertion of transcriptional terminators T*_las_*, T*_rps9_*, and T*_leuB_* unexpectedly facilitated excision of the Sp^R^ gene in the transformants. In contrast, a modified stem-loop of T*_clpP_* (pBFK86) increased the proportion of Sp^R^ transformants to 98.9%, indicating the ability of the potent transcriptional terminator T*_clpP_* to decrease expression of the promoterless Cre gene. Even after 41 generations of culture without Sp, 97.5% of pBFK86-carrying clones (731/750 clones) retained Sp^R^. Furthermore, 99.7% of the clones (395/396 clones) showed the Sp^R^ phenotype when this strain was grown in Sp-containing medium, administered orally to mice fed a standard chow diet, and then recovered from feces at 12 h after administration. 

We next analyzed whether insertion of the raffinose-inducible promoter P*_cscBA_* into pBFK86 increased the excision rate of the Sp^R^ gene (Figure 2). Generated plasmid pBFK94 was introduced into the *loxP*-Sp strain and used for the analysis. When the transformants were incubated on 1/2 MRSCS-Cm agar plates containing glucose or raffinose as the sole carbohydrate source, 86.6% of clones (175/202 clones) showed Sp^R^ on glucose (uninduced condition). In contrast, only 10.1% of clones (28/278 clones) retained Sp^R^ on raffinose (induced condition). These results strongly suggested that pBFK86 was suitable as the promoterless Cre plasmid of the R-IVET system for *B. longum* 105-A. 

### 3.2. Construction of the Genomic DNA Library

The R-IVET library consisting of ~120,000 clones was constructed by inserting the genomic DNA fragment from *B. longum* 105-A upstream of the promoterless Cre gene in pBFK86 and introducing them into the *loxP*-Sp strain. Colony PCR showed that the 78 tested strains harbored different sizes of the inserted DNA fragments. DNA sequencing further revealed that (i) 77.5% of clones had a single DNA fragment, (ii) all DNA fragments were unique, and (iii) the average fragment length was 787 bp. Based on the average size of the inserted fragment, it was estimated that 20,098 clones were necessary to cover 99.9% of the 2.3 Mbp of *B. longum* 105-A genomic DNA (Clarke and Carbon formula [40]). These results indicated that the quality and coverage of the library were sufficient for further analyses. It should be noted that only 60.0% of clones in the library retained the Sp^R^ gene, indicating that various inserted DNA fragments were functional as a promoter under in vitro conditions.

### 3.3. Screening of In Vivo-Induced Genes

#### 3.3.1. First and Second Trials

**Figure 3:**
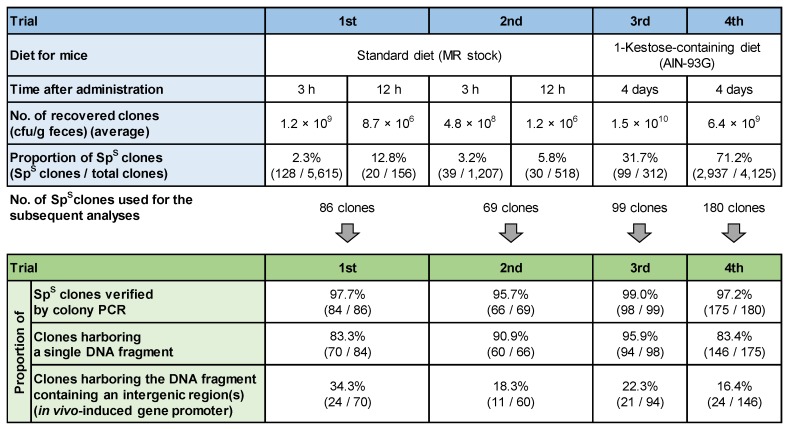
Summary of the results leading to identification of candidate in vivo-induced genes in the four trials of the R-IVET analysis. First and second trials were performed with four mice, respectively. Third and fourth trials were carried out with two mice, respectively. See Section 2.2 and Section 2.7 of the Materials and Methods for detailed procedures.

The first and second trials were carried out using mice fed a standard chow diet (see Section 2.7.1) (Figure 3). Abundant clones were recovered from feces at concentrations of 10^8–9^ cfu/g feces at 3 h after administration. In contrast, the concentration was rapidly decreased to 10^6^ cfu/g feces at 12 h after administration, indicating a low persistence of *B. longum* 105-A in the intestinal tract of mice. The *B. longum* 105-A clones collected from mice feces at 3 and 12 h after administration showed the Sp^S^ phenotype at proportions of 2.3% (128/5615 clones) and 12.8% (20/156 clones), respectively, at the first trial and 3.2% (39/1207 clones) and 5.8% (30/518 clones) at the second trial. Then, 155 clones (86 clones from the first trial and 69 clones from the second trial) were used for further analyses. Colony PCR analysis revealed excision of the Sp^R^ gene in 84 out of 86 clones in the first trial and 66 out of 69 clones in the second trial. Sanger sequencing showed insertion of a single and unique DNA fragment upstream of the Cre gene in 70 strains in the first trial and 60 strains in the second trial. Among them, 24 and 11 strains, respectively, harbored DNA fragments containing an intergenic region(s) (candidate in vivo-induced gene promoter) located in the same direction as the Cre gene (Table 3). Genes located downstream of the candidate promoter in *B. longum* 105-A genome were selected as in vivo-induced genes in these two trials.

#### 3.3.2. Third and Fourth Trials

The third and fourth trials were conducted using mice fed the 1-kestose-containing diet to promote intestinal colonization of *B. longum* 105-A (see Section 2.7.2). In these trials, the clones were recovered at high concentrations (10^9–10^ cfu/g feces) even at 4 days after administration (Figure 3), indicating the increased persistence of *B. longum* 105-A in the mouse intestines by feeding the 1-kestose-containing diet. The proportion of Sp^S^ clones in the recovered colonies at 4 days after administration was increased to 31.7% (99/312 clones) and 71.2% (2937/4125 clones), respectively. An obvious increase in the proportions of Sp^S^ clones compared with the first and second trials (Section 3.3.1) also reflects the prolonged colonization of the administrated strain in the mouse intestines. Next, 99 clones from the third trial and 180 clones from the fourth trial were used for further analysis. Colony PCR analysis revealed excision of the Sp^R^ gene in 98 out of 99 clones of the third trial and 175 out of 180 clones in the fourth trial. Sanger sequencing showed insertion of a single and unique DNA fragment upstream of the Cre gene in 94 strains in the third trial and 146 strains in the fourth trial. Among them, 21 and 24 strains, respectively, harbored DNA fragments containing an intergenic region(s) (candidate in vivo-induced gene promoter) located in the same direction as the Cre gene (Table 3). Considering redundant detection of candidate genes in the four rounds of administration experiments, 73 different genes, which were assigned to various COG categories, were finally identified as in vivo-induced genes (Table 3). 

### 3.4. Verification of In Vivo-Induced Gene Expression in the Cecum

**Figure 4:**
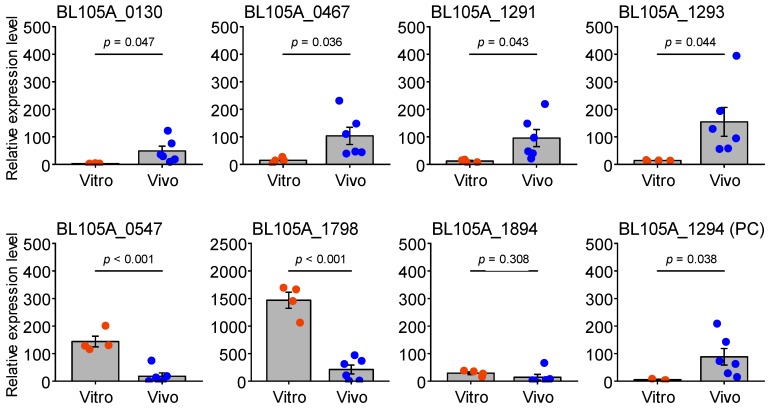
In vitro and in vivo relative expression levels of the genes identified by R-IVET. BL105A_1294 (encoding β-fructofuranosidase (glycoside hydrolase family 32)) was used as a positive control (PC) in qRT-PCR analysis, while the other genes were used to verify in vivo-induced expression in the cecum. BL105A_1946 (*rnpA*) was used as a reference gene. Data obtained from in vitro (*n* = 4) and in vivo (*n* = 6) conditions are expressed as the mean ± standard deviation together with each data plot. After testing the equality of variance by the *F*-test, Student’s or Welch two-tailed *t*-tests were used to evaluate statistical significance. *p*-values of the *t*-tests are also indicated in each panel and *p* < 0.05 was considered as statistically significant.

Among the 73 genes identified by R-IVET, in vivo-induced expression of selected genes was verified by comparing the gene expression of *B. longum* 105-A in vitro (cultured in 1/2MRSCS-Cm) and in vivo (in mouse cecal contents) using qRT-PCR (Figure 4). In vivo-induced expression of the positive control gene, BL105A_1294 (β-fructofuranosidase), indicated that the qRT-PCR analysis conducted in this study was rational for the verification. The qRT-PCR analysis verified in vivo-induced expression of four out of seven tested genes in the cecum. In vivo-induced expression of BL105A_0467 (putative adhesin) and BL105A_1291 (serine protease inhibitor) was verified as observed in the transcriptome analysis of *B. breve* UCC2003 colonized in conventionally raised BALB/c mice [17]. BL105A_0130 (presumable pilin subunit for Tad pili) was also significantly induced in vivo as inferred by the induced expression of the Tad pilus-encoding gene cluster in *B. breve* UCC2003 colonized in BALB/c mice [17]. BL105A_1293 (galactoside transport protein) was also confirmed as an in vivo-induced gene. Collectively, these results indicated that R-IVET is a rational strategy to identify in vivo-induced genes of *B. longum*.

## 4. Discussion

This study demonstrated the novel application of R-IVET to genus *Bifidobacterium* and identified 73 in vivo-induced genes in *B. longum* 105-A. As shown in Section 3.3, several genes were commonly identified to be induced in the digestive tract of the conventionally raised BALB/c mice colonized with *B. longum* 105-A (R-IVET) or *B. breve* UCC2003 (DNA microarray) (Table 3) [17]. However, the obtained gene dataset showed overlapping yet different contents compared with the transcriptome dataset of *B. breve* UCC2003 [17]. Although they are not appropriately comparable because different *Bifidobacterium* species/strains were used in these studies, the observed difference in the gene datasets appears to be partially attributed to the distinct principles of R-IVET and DNA microarray approaches. R-IVET evaluates in vivo expression in each single cell by an irreversible recombination reaction. Therefore, this technology enables identification of transiently and site-specifically expressed genes. In contrast, these genes are difficult to detect by other transcriptome approaches, such as DNA microarray and RNA-sequencing, in principle because these technologies evaluate average gene expression levels of multiple cells of the bacteria in a given environment. R-IVET also generates in vivo gene expression data in the presence of the gut microbiota irrespective of the persistence ability of the bacterial strains in certain environments. RNA-sequencing is not suitable to analyze the in vivo gene expression of low-persistent strains because huge numbers of reads are required to obtain sufficient information on the transcripts of target strains. DNA microarray may be hindered by unexpected cross-hybridization with cDNA from closely related bacteria. In contrast to the advantages, R-IVET disadvantageous because (i) comprehensive gene identification is not feasible owing to the tedious and complicated procedures, (ii) application of R-IVET is limited to genetically amenable strains, and (iii) further analyses are required to identify sites of in vivo gene expression. Taken together, the R-IVET data obtained in this study provide valuable information for comprehensive understanding of the in vivo commensal mechanisms of *B. longum*, especially when integrated with other types of transcriptomic data.

In this study, we verified the in vivo-induced expression of seven genes identified by the R-IVET analysis using qRT-PCR. Four genes (BL105A_0130, BL105A_0467, BL105A_1291, and BL105A_1293) showed significantly increased expression in the cecum, but the other three genes (BL105A_0547, BL105A_1798, and BL105A_1894) did not (Figure 4). Although this inconsistency might be attributed to that different mice were used between these two assays, there are some other possible reasons. The first possible reason is that the in vivo-induction does not necessarily occur in the cecum. Indeed, several in vivo-induced genes in *Lactobacillus plantarum* WCFS1, which was identified by R-IVET as conducted in this study, are reported to be expressed transiently or locally in the gastrointestinal tract other than in the cecum [41]. This finding suggests that the three genes detected in our R-IVET analysis might be also expressed in the similar pattern. 

The second possible reason is the occurrence of false-positive clones lacking the promoter region (discussed later) due to the high sensitivity of the Cre/*loxP* system used in this study. The sensitivity of the Cre/*loxP* system in *B. longum* 105-A was so high that it induced excision of the Sp^R^ gene even by a slight level of Cre expression from the promoterless Cre expression vectors (Figure 2). The high sensitivity was also indicated by comparing with the reported results of R-IVET in *L. plantarum* WCFS1, which adopted the almost same strategy as this study [19]. It is evident from the rates of the loss of the antibiotic-resistance gene in the libraries from the two studies: (i) 40% in *B. longum* and 10% in *L. plantarum* during preparation of the genomic DNA library; (ii) 2.8% at 3 h and 9.3% at 12 h after administration of *B. longum* (average data of the first and second trials) compared with 3.3% even at 24 h after administration of *L. plantarum*, when the library was recovered from feces (Figure 3). High sensitivity of the Cre/*loxP* system in *B. longum* 105-A is advantageous for detection of genes that are not expressed in vitro, but induced in vivo at a low level although it may be concurrently disadvantageous because of the detection of the false-positive clones. 

Our R-IVET used a random genomic library constructed by partial digestion of the genomic DNA of *B. longum* 105-A with Sau3AI. Therefore, the inserted sequence was not always incorporated into the clone as the promoter exists to allow expression of Cre. In other words, clones harboring a truncated promoter region (digested within the promoter by Sau3AI) or without harboring a promoter region would be generated. We examined the putative vegetative promoter sequence in the inserted sequence of R-IVET clones corresponding to the seven genes (Supplementary Figure S1). As expected, clones for four genes, in which in vivo-induced expression were validated by qRT-PCR analysis, harbored the 5′ region of the ORF and its upstream region in the inserted sequences. Furthermore, the promoter regions deduced from the consensus sequence of the bacterial vegetative promoter (5′-TATAAT-3′ as the −10 region and 5′-TTGACA-3′ as the −35 region), which was detected in the genome of *B. breve* UCC2003 [42], were predicted at the 20–249 bp upstream position from the 5′ end of the ORF (Supplementary Figure S1). The predicted positions of these promoters from the ORF were consistent with the length of the 5′ untranslated region (7–240 bp) of the genes in the *B. breve* UCC2003 genome [42]. Consequently, consistent results of the expression of the four genes in R-IVET and qRT-PCR analyses were thought to be caused by possible Cre expression in the clones as occurred in the *B. longum* 105-A genome. By contrast, the corresponding clones of two out of three genes, which showed inconsistent results regarding the gene expression R-IVET and qRT-PCR analyses, did not harbor the predictable promoter sequence (Supplementary Figure S1). These genes were possibly detected in R-IVET as a false positive due to the high sensitivity of our Cre/*loxP* system. The inserted sequence of the clone for BL105A_1798 showed a similar DNA structure to those observed in the four genes showing consistent expression (Supplementary Figure S1). Transient expression or local expression in other than the cecum might have occurred as mentioned above (the first possible reason). 

Detection of the false-positive clones is an issue in the current R-IVET system in *B. longum* 105-A. Further repeated R-IVET trials together with the use of the increased numbers of mice will improve the reliability of the in vivo-induced genes identified by R-IVET. Additional verification of the in vivo-induction of the identified genes by qRT-PCR will be also necessary. From another perspective, further improvement of the current R-IVET system will be effective to obtain more reliable data. One effective solution to reduce the false-positive clones is decreasing the sensitivity of Cre/*loxP* by modifying the *loxP* sequence. In fact, in the study of *Enterococcus faecalis*, fewer genes were identified by R-IVET using mutated *loxP* than that using native *loxP* [22].

Although there remains some future issues, R-IVET adopted in this study is an attractive approach to identify *B. longum* genes induced in the gastrointestinal tract of mice. Combining R-IVET with other transcriptome analyses based on the different principles may lead to more in-depth understanding the strategies of *B. longum* for survival and colonization in the intestinal tract. As a further step, we are aiming to reveal the functions and physiological roles of in vivo-induced genes by gene disruption approaches. Such analyses may provide further significant information to reveal the in vivo commensal mechanisms of *B. longum*.

## Figures and Tables

**Table 1:** Representative bacterial strains used in this study.

Strain	Description ^1^	Source or Reference
***Escherichia coli***		
*E. coli* DH5α	F^−^, Φ80d *lacZ*ΔM15, Δ(*lacZYA-argF*) U169, *deoR*, *recA*1, *endA*1, *hsdR*17(r_K_^−^ m_K_^+^), *phoA*, *supE*44, λ^−^, *thi*-1, *gyrA*96, *relA*1	National BioResource Project (NIG, Mishima, Japan)
***Bifidobacterium***		
*B. longum* subsp. *longum* 105-A (JCM 31944)	Human fecal isolate	[25]
*loxP*-Sp strain	*B. longum* 105-A derivative strain harboring *loxP*-Sp^R^-*loxP* cassette on the chromosome, Sp^R^	This study

^1^ Sp^R^: spectinomycin resistance.

**Table 2:** Primers and DNA templates used in this study.

No.	PCR Product ^1^	DNA Template	Cloning Strategy ^2^	Primer	Nucleotide Sequence (5′-3′) ^3^
**Integration of *loxP*-Sp^R^-*loxP* into a chromosome of *B. longum* 105-A (construction of *loxP*-Sp strain)**					
1	Sp^R^ gene	pBS423 [27]	Blunt-end ligation	Pr-Blo0041	GCATGCCTGCAGGTCGATTTTC
				Pr-Blo0042	CAAAAAAATTGAAAAAAGTGTTTCCAC
2	Homologous region to BL105A_1451 locus (HR1)	*B. longum* 105-A genomic DNA	Restriction-ligation	Pr-Blo0100	GCGAATTCATGACGTACGATTTCACGTCG
				Pr-Blo0101	TAGAATTCCGCAATCGCGGATGCATGCCGT
3	Homologous region to BL105A_1452 locus (HR2)	*B. longum* 105-A genomic DNA	Restriction-ligation	Pr-Blo0098	AGGGATCCGTGTCCTGGAAAGACGAATGCC
				Pr-Blo0099	TGGGATCCATGTCCGTTTCGCAGTCACCGG
4	HR1-*loxP*-Sp^R^-*loxP*-HR2	pBFH35 (this study)	In-Fusion cloning	Pr-Blo0119	TATATATGAGTACTGAGGTCGACTCTAGAGGATCC
				Pr-Blo0120	AAACGACGGCCAGTTAAACGACGGCCAGTGAATTG
5	pBS423Δ*repA* lacking Sp^R^ gene	pBFS423Δ*repA* [27]	In-Fusion cloning	Pr-Blo0116	CAGTACTCATATATACTTTAGATTGATTTA
				Pr-Blo0117	AAGCTTGCATGCCTGCAGATAGGCAG
**Construction of Cre expression plasmid**					
6	Cm^R^ gene	pBFS38 [29]	In-Fusion cloning	Pr-Blo0239	AAAGTATATATGAGTACTTGGGCGCGGCGGCCATGAAG
				Pr-Blo0240	GCGGCCGCGCCGGCATGCATTATGGAAGCGCTGAACTAGTC
7	BglII-RBS_h4_-Cre ORF	Bacteriophage P1 genomic DNA	In-Fusion cloning	Pr-Blo0247	CTTCCCGGCGAGATCCTAATCGCCATCTTCCAGC
				Pr-Blo0249	GATTACTTCGGCGCGAGATCTCCCAAGAAGGATGCTATGTCCAATTTACTGACCGTACAC
8	BglII-RBS_h3_-Cre ORF	Bacteriophage P1 genomic DNA	In-Fusion cloning	Pr-Blo0247	CTTCCCGGCGAGATCCTAATCGCCATCTTCCAGC
				Pr-Blo0257	GATTACTTCGGCGCGAGATCTCCCAAGAAGGATGCATGTCCAATTTACTGACCGTACAC
9	T*_las_*	*L. lactis* subsp. *cremoris* MG1363 genomic DNA	Restriction-ligation	Pr-Blo0258	ACGTGGATCCGGACAATATGGGGTAAGCG
				Pr-Blo0259	AAGAAGATCTCTAAAGCTGACGGGGTAAAC
10	T*_rps9_*	*B. longum* 105-A genomic DNA	Restriction-ligation	Pr-Blo0264	TGACCGAGATCTTGTGGATGATACACCGGACACTC
				Pr-Blo0265	TAGGGATCCTCGTGGAGCGCAAGAAGGCTGGTCTG
11	T*_leuB_*	*C. glutamicum* ATCC 13032 genomic DNA	Restriction-ligation	Pr-Blo0260	GTATGCAGATCTCCAGCAAGTATTTACACCAAC
				Pr-Blo0261	AGTGGATCCTGCGATGCTGCTGCGTCACTTAG
12	T*_clpP_* stem loop-BglII-RBS_h3_-Cre ORF	pBFK71 (this study)	In-Fusion cloning	Pr-Blo0277	ATGGCTTCCCGGCGACTAATCGCCATCTTCCAGC
				Pr-Blo0280	ATTACTTCGGCGCGAaaaaccctcggtcggtctgaccgggggttttAGATCTCCCAAGAAGGATGCATG
13	P*_cscBA_*	*B. longum* 105-A genomic DNA	Restriction-ligation	Pr-Blo0292	ATTAGATCTTTGGTTGGTTATTGGTTATGTAAC
				Pr-Blo0293	ATTAGATCTCCGAGTCCCACACGATTTCTC
**Genotypic analysis of the *loxP*-Sp strain**					
14	Sp^R^ gene	Genomic DNA of *loxP*-Sp strain	NA	Pr-Blo0099	TGGGATCCATGTCCGTTTCGCAGTCACCGG
				Pr-Blo0100	GCGAATTCATGACGTACGATTTCACGTCG
**Determination of Inserted DNA Fragments in the R-IVET Library**					
15	Inserted DNA fragment	pBFK86 derivative carrying a random DNA fragment (this study)	NA	Pr-Blo0277	ATGGCTTCCCGGCGACTAATCGCCATCTTCCAGC
				Pr-Blo0318	GTAAGCGGCAGGGTCGGAACAGGAGAGCG
**qRT-PCR analysis**					
16	BL105A_0130	*B. longum* 105-A genomic DNA	NA	Pr-Blo0414	AGGCGAAAGAACGGCTATGC
				Pr-Blo0415	GACTTCAGGATGGCGACCAG
17	BL105A_0467	*B. longum* 105-A genomic DNA	NA	Pr-Blo0416	CCTTGTTGCCCAGACCCAAC
				Pr-Blo0417	CATAAGAGCGACGCAGCGAG
18	BL105A_0547	*B. longum* 105-A genomic DNA	NA	Pr-Blo0432	TCGGCAACCATGTTGAGCAC
				Pr-Blo0433	GCCTACCCCGATCAGCTCTC
19	BL105A_1291	*B. longum* 105-A genomic DNA	NA	Pr-Blo0434	ATGTTCAAGCCGAAGGCCAC
				Pr-Blo0435	GCCATCCACATCGAAGCAGG
20	BL105A_1293	*B. longum* 105-A genomic DNA	NA	Pr-Blo0436	AAATCGGCAACGCCACCTAC
				Pr-Blo0437	CGCAGGAACATCACGGTAGC
21	BL105A_1294	*B. longum* 105-A genomic DNA	NA	Pr-Blo0408	AAGGTCGACCACCACTACCG
				Pr-Blo0409	CTCGTATTCCCAGCGGACCA
22	BL105A_1798	*B. longum* 105-A genomic DNA	NA	Pr-Blo0428	GCATCGCGGGAAGAACAGAC
				Pr-Blo0429	ATACGCAAACGGCTTCACCG
23	BL105A_1894	*B. longum* 105-A genomic DNA	NA	Pr-Blo0430	CCACCGACGACCCACTTTTG
				Pr-Blo0431	AGTCGAACCAGACCATCCCG
24	BL105A_1946	*B. longum* 105-A genomic DNA	NA	Pr-Blo0372	GCCTTCGCGATCTGCTGATCTAG
				Pr-Blo0373	ACCCGTAATACGGTGAAGCGTAG

^1^ Sp^R^: spectinomycin resistance, Cm^R^: chloramphenicol resistance; ^2^ NA: not applied; ^3^ Bold single underlines indicate restriction sites, while normal single underlines represent the sequences for In-Fusion cloning. Double underlines indicate the ribosome-binding site (RBS) and spacer region. Lowercase letters represent the sequences for the modified T*_clpP_* stem-loop.

**Table 3:** In vivo-induced genes identified by R-IVET using *B. longum* 105-A.

No.	In Vivo Induced Genes ^1^	Annotation ^1^	Identified Round	COG Category ^2, 3^
1	BL105A_0064	Hypothetical protein	2nd	–
2	BL105A_0075	Hypothetical protein	3rd	S
3	BL105A_0117	GrpE protein	1st	O
4	BL105A_0130	Presumable pilin subunit for the Tad-pili	4th	–
5	BL105A_0136	Recombination protein RecR	1st	L
6	BL105A_0138	Hypothetical protein	4th	–
7	BL105A_0202	ABC transporter permease component	4th	G
8	BL105A_0204	Glycoside hydrolase family 127 β-l-arabinofuranosidase	4th	S
9	BL105A_0248	Hypothetical protein	3rd	–
10	BL105A_0262	Hypothetical protein	4th	–
11	BL105A_0267	Hypothetical protein	1st, 2nd, 4th	–
12	BL105A_0338	Ribonuclease VapC	4th	R
13	BL105A_0374	Magnesium-translocating P-type ATPase	4th	–
14	BL105A_0377	Hypothetical protein	1st	–
15	BL105A_0414	Oligosaccharide repeat unit polymerase Wzy	2nd	M
16	BL105A_0415	Hypothetical protein	4th	M
17	BL105A_0422	Transposase	4th	X
18	BL105A_0423	Integrase catalytic region	1st	X
19	BL105A_0467	Putative adhesin	3rd	X, R
20	BL105A_0490	Putative ABC transporter ATP-binding component	3rd	E
21	BL105A_0507	Peptides ABC transporter ATP-binding component	1st	P, E
22	BL105A_0534	Hypothetical protein	3rd	V, M
23	BL105A_0540	Hypothetical protein	3rd	V
24	BL105A_0547	ATPase of the ABC transporter	3rd, 4th	E
25	BL105A_0662	Transcriptional regulator	2nd	M
26	BL105A_0669	Putative phosphoribosylpyrophosphate amidotransferase	3rd	R
27	BL105A_0776	Hypothetical protein	3rd, 4th	–
28	BL105A_0812	Shikimate kinase/3-dehydroquinate synthase	4th	E
29	BL105A_0835	NAD(P) transhydrogenase α-2 subunit	2nd	C
30	BL105A_0854	Hypothetical protein	2nd	V
31	BL105A_0900	Hypothetical protein	3rd	–
32	BL105A_0929	Hypothetical protein	1st	–
33	BL105A_0934	Phosphoribosyl-ATP pyrophosphatase	2nd	E
34	BL105A_1028	Hypothetical protein	3rd	–
35	BL105A_1049	Hypothetical protein	1st	–
36	BL105A_1053	Hypothetical protein	4th	–
37	BL105A_1079	tRNA N6-adenosine threonylcarbamoyltransferase	1st	J
38	BL105A_1118	Hypothetical protein	1st	–
39	BL105A_1123	RecX-like protein	3rd	O
40	BL105A_1233	Cell division protein FtsW	3rd	D
41	BL105A_1250	16S RNA methylase	1st	J
42	BL105A_1253	Transporter	2nd	G
43	BL105A_1291	Serine protease inhibitor	1st	O
44	BL105A_1293	Galactoside transport protein	1st	P
45	BL105A_1371	ABC-type fructose transport system ATPase subunit FruK	4th	G
46	BL105A_1419	Hypothetical protein	3rd	I
47	BL105A_1426	Hypothetical protein	4th	–
48	BL105A_1456	Sugar kinase in PfkB family	4th	G, F
49	BL105A_1489	Endonuclease	4th	L
50	BL105A_1517	Peptide chain release factor 1	4th	J
51	BL105A_1556	Hypothetical protein	4th	N
52	BL105A_1562	tRNA-Phe	3rd	–
53	BL105A_1583	Hypothetical protein	3rd	–
54	BL105A_1603	Sugar ABC transporter permease component	2nd	G
55	BL105A_1605	Hypothetical protein	1st	–
56	BL105A_1637	DNA-directed RNA polymerase α subunit	1st	K
57	BL105A_1680	Amino acid transporter	1st	E
58	BL105A_1696	Hypothetical protein	4th	L
59	BL105A_1707	Possible extracellular *exo*-xylanase	4th	G
60	BL105A_1708	*endo*-1,4-β-Xylanase	2nd	G
61	BL105A_1718	Hypothetical protein	1st	G
62	BL105A_1733	16S ribosomal RNA	1st	–
63	BL105A_1798	Putative glycosyltransferase	1st, 3rd	M
64	BL105A_1810	Probable potassium uptake protein Kup	3rd	P
65	BL105A_1828	Hypothetical protein	1st	–
66	BL105A_1834	Hypothetical protein	1st, 1st	L
67	BL105A_1857	Hypothetical protein	4th	R, G
68	BL105A_1883	α-Glucosidase	3rd	G
69	BL105A_1885	Glycosidase	1st	G
70	BL105A_1886	Permease protein of ABC transporter system for sugars	4th	G
71	BL105A_1894	Raffinose transport system permease protein	2nd, 3rd	G
72	BL105A_1910	Lipopolysaccharide kinase	3rd	T
73	BL105A_1945	Preprotein translocase subunit YidC	1st	M

^1^ The complete genome sequence of *B. longum* 105-A (GenBank accession no. AP014658.1) [36] was used as a reference. ^2^ [J] Translation, ribosomal structure and biogenesis; [A] RNA processing and modification; [K] Transcription; [L] Replication, recombination, and repair; [B] Chromatin structure and dynamics; [D] Cell cycle control, cell division, chromosome partitioning; [Y] Nuclear structure; [V] Defense mechanisms; [T] Signal transduction mechanisms; [M] Cell wall/membrane/envelope biogenesis; [N] Cell motility; [Z] Cytoskeleton; [W] Extracellular structures; [U] Intracellular trafficking, secretion, and vesicular transport; [O] Post-translational modification, protein turnover, chaperones; [X] Mobilome: prophages, transposons; [C] Energy production and conversion; [G] Carbohydrate transport and metabolism; [E] Amino acid transport and metabolism; [F] Nucleotide transport and metabolism; [H] Coenzyme transport and metabolism; [I] Lipid transport and metabolism; [P] Inorganic ion transport and metabolism; [Q] Secondary metabolite biosynthesis, transport and catabolism; [R] General function prediction only; [S] Function unknown. ^3^ –: not assigned into COG categories.

## Supplementary Materials

The following are available online at https://www.mdpi.com/2076-2607/8/3/410/s1, Figure S1: The structure and genomic position of the inserted DNA fragment in representative R-IVET clones, Table S1: Diet compositions for the third and fourth R-IVET experiments.

## References

[1] Nishijima S., Suda W., Oshima K., Kim S.W., Hirose Y., Morita H., Hattori M. (2016). The gut microbiome of healthy Japanese and its microbial and functional uniqueness. DNA Res..

[2] Odamaki T., Kato K., Sugahara H., Hashikura N., Takahashi S., Xiao J.Z., Abe F., Osawa R. (2016). Age-related changes in gut microbiota composition from newborn to centenarian: A cross-sectional study. BMC Microbiol..

[3] Sakanaka M., Hansen M.E., Gotoh A., Katoh T., Yoshida K., Odamaki T., Yachi H., Sugiyama Y., Kurihara S., Hirose J. (2019). Evolutionary adaptation in fucosyllactose uptake systems supports bifidobacteria-infant symbiosis. Sci. Adv..

[4] Mattarelli P., Biavati B., Mattarelli P., Biavati B., Holzapfel W.H., Wood B.J.B. (2018). Species in the genus Bifidobacterium. The Bifidobacteria and Related Organisms: Biology, Taxonomy, Applications.

[5] Kato K., Odamaki T., Mitsuyama E., Sugahara H., Xiao J.Z., Osawa R. (2017). Age-related changes in the composition of gut *Bifidobacterium* species. Curr. Microbiol..

[6] Odamaki T., Bottacini F., Kato K., Mitsuyama E., Yoshida K., Horigome A., Xiao J.Z., van Sinderen D. (2018). Genomic diversity and distribution of *Bifidobacterium longum* subsp. *longum* across the human lifespan. Sci. Rep..

[7] Milani C., Mangifesta M., Mancabelli L., Lugli G.A., James K., Duranti S., Turroni F., Ferrario C., Ossiprandi M.C., van Sinderen D. (2017). Unveiling bifidobacterial biogeography across the mammalian branch of the tree of life. ISME J..

[8] Hidalgo-Cantabrana C., Delgado S., Ruiz L., Ruas-Madiedo P., Sánchez B., Margolles A. (2017). Bifidobacteria and their health-promoting effects. Microbiol. Spectr..

[9] Leahy S.C., Higgins D.G., Fitzgerald G.F., van Sinderen D. (2005). Getting better with bifidobacteria. J. Appl. Microbiol..

[10] Fujita K., Sasaki Y., Kitahara K. (2019). Degradation of plant arabinogalactan proteins by intestinal bacteria: Characteristics and functions of the enzymes involved. Appl. Microbiol. Biotechnol..

[11] Katayama T. (2016). Host-derived glycans serve as selected nutrients for the gut microbe: Human milk oligosaccharides and bifidobacteria. Biosci. Biotechnol. Biochem..

[12] Suzuki K., Nishiyama K., Miyajima H., Osawa R., Yamamoto Y., Mukai T. (2016). Adhesion properties of a putative polymorphic fimbrial subunit protein from *Bifidobacterium longum* subsp. longum. Biosci. Microbiota Food Health.

[13] McCarville J.L., Dong J., Caminero A., Bermudez-Brito M., Jury J., Murray J.A., Duboux S., Steinmann M., Delley M., Tangyu M. (2017). A commensal *Bifidobacterium longum* strain prevents gluten-related immunopathology in mice through expression of a serine protease inhibitor. Appl. Environ. Microbiol..

[14] Sonnenburg J.L., Chen C.T.L., Gordon J.I. (2006). Genomic and metabolic studies of the impact of probiotics on a model gut symbiont and host. PLoS Biol..

[15] Sugahara H., Odamaki T., Fukuda S., Kato T., Xiao J.Z., Abe F., Kikuchi J., Ohno H. (2015). Probiotic *Bifidobacterium longum* alters gut luminal metabolism through modification of the gut microbial community. Sci. Rep..

[16] Ishikawa E., Shima T., Suda K., Shirasawa Y., Sato T., Umesaki Y. (2011). Comparison of *Bifidobacterium breve* strain Yakult transcriptomes in germ-free mice with those in fecal cultures. J. Biosci. Bioeng..

[17] O’Connell-Motherway M., Zomer A., Leahy S.C., Reunanen J., Bottacini F., Claesson M.J., O’Brien F., Flynn K., Casey P.G., Munoz J.A.M. (2011). Functional genome analysis of *Bifidobacterium breve* UCC2003 reveals type IVb tight adherence (Tad) pili as an essential and conserved host-colonization factor. Proc. Natl. Acad. Sci. USA.

[18] O’Connell-Motherway M., O’Brien F., O’Driscoll T., Casey P.G., Shanahan F., van Sinderen D. (2018). Carbohydrate syntrophy enhances the establishment of *Bifidobacterium breve* UCC2003 in the neonatal gut. Sci. Rep..

[19] Bron P.A., Grangette C., Mercenier A., de Vos W.M., Kleerebezem M. (2004). Identification of *Lactobacillus plantarum* genes that are induced in the gastrointestinal tract of mice. J. Bacteriol..

[20] Bachmann H., Kleerebezem M., van Hylckama Vlieg J.E.T. (2008). High-throughput identification and validation of *in situ*-expressed genes of *Lactococcus lactis*. Appl. Environ. Microbiol..

[21] Bachmann H., de Wilt L., Kleerebezem M., van Hylckama Vlieg J.E.T. (2010). Time-resolved genetic responses of *Lactococcus lactis* to a dairy environment. Environ. Microbiol..

[22] Hanin A., Sava I., Bao Y.Y., Huebner J., Hartke A., Auffray Y., Sauvageot N. (2010). Screening of *in vivo* activated genes in *Enterococcus faecalis* during insect and mouse infections and growth in urine. PLoS ONE.

[23] Junjua M., Galia W., Gaci N., Uriot O., Genay M., Bachmann H., Kleerebezem M., Dary A., Roussel Y. (2014). Development of the recombinase-based *in vivo* expression technology in *Streptococcus thermophilus* and validation using the lactose operon promoter. J. Appl. Microbiol..

[24] Uriot O., Galia W., Awussi A.A., Perrin C., Denis S., Chalancon S., Lorson E., Poirson C., Junjua M., Le Roux Y. (2016). Use of the dynamic gastro-intestinal model TIM to explore the survival of the yogurt bacterium *Streptococcus thermophilus* and the metabolic activities induced in the simulated human gut. Food Microbiol..

[25] Matsumura H., Takeuchi A., Kano Y. (1997). Construction of *Escherichia coli–Bifidobacterium longum* shuttle vector transforming *B. longum* 105-A and 108-A. Biosci. Biotechnol. Biochem..

[26] De Man J.C., Rogosa M., Sharpe M.E. (1960). A medium for the cultivation of lactobacilli. J. Appl. Bacteriol..

[27] Hirayama Y., Sakanaka M., Fukuma H., Murayama H., Kano Y., Fukiya S., Yokota A. (2012). Development of a double-crossover markerless gene deletion system in *Bifidobacterium longum*: Functional analysis of the α-galactosidase gene for raffinose assimilation. Appl. Environ. Microbiol..

[28] Kanegae Y., Lee G., Sato Y., Tanaka M., Nakal M., Sakaki T., Sugano S., Saito I. (1995). Efficient gene activation in mammalian cells by using recombinant adenovirus expressing site-specific Cre recombinase. Nucleic Acids Res..

[29] Sakanaka M., Tamai S., Hirayama Y., Onodera A., Koguchi H., Kano Y., Yokota A., Fukiya S. (2014). Functional analysis of bifidobacterial promoters in *Bifidobacterium longum* and *Escherichia coli* using the α-galactosidase gene as a reporter. J. Biosci. Bioeng..

[30] Yasui K., Kano Y., Tanaka K., Watanabe K., Shimizu-Kadota M., Yoshikawa H., Suzuki T. (2009). Improvement of bacterial transformation efficiency using plasmid artificial modification. Nucleic Acids Res..

[31] He J., Sakaguchi K., Suzuki T. (2012). Determination of the ribosome-binding sequence and spacer length between binding site and initiation codon for efficient protein expression in *Bifidobacterium longum* 105-A. J. Biosci. Bioeng..

[32] Sternberg N., Sauer B., Hoess R., Abremski K. (1986). Bacteriophage P1 *cre* gene and its regulatory region. Evidence for multiple promoters and for regulation by DNA methylation. J. Mol. Biol..

[33] Llanos R.M., Harris C.J., Hillier A.J., Davidson B.E. (1993). Identification of a novel operon in *Lactococcus lactis* encoding three enzymes for lactic acid synthesis: Phosphofructokinase, pyruvate kinase, and lactate dehydrogenase. J. Bacteriol..

[34] Pátek M., Hochmannová J., Jelínková M., Nešvera J., Eggeling L. (1998). Analysis of the *leuB* gene from *Corynebacterium glutamicum*. Appl. Microbiol. Biotechnol..

[35] Ruiz L., O’Connell-Motherway M., Lanigan N., van Sinderen D. (2013). Transposon mutagenesis in *Bifidobacterium breve*: Construction and characterization of a Tn*5* transposon mutant library for *Bifidobacterium breve* UCC2003. PLoS ONE.

[36] Kanesaki Y., Masutani H., Sakanaka M., Shiwa Y., Fujisawa T., Nakamura Y., Yokota A., Fukiya S., Suzuki T., Yoshikawa H. (2014). Complete genome sequence of *Bifidobacterium longum* 105-A, a strain with high transformation efficiency. Genome Announc..

[37] Galperin M.Y., Makarova K.S., Wolf Y.I., Koonin E.V. (2015). Expanded microbial genome coverage and improved protein family annotation in the COG database. Nucleic Acids Res..

[38] Sakanaka M., Nakakawaji S., Nakajima S., Fukiya S., Abe A., Saburi W., Mori H., Yokota A. (2018). A transposon mutagenesis system for *Bifidobacterium longum* subsp. *longum* based on an IS3 family insertion sequence, *ISBlo11*. Appl. Environ. Microbiol..

[39] Tanno H., Fujii T., Ose R., Hirano K., Tochio T., Endo A. (2019). Characterization of fructooligosaccharide-degrading enzymes in human commensal *Bifidobacterium longum* and *Anaerostipes caccae*. Biochem. Biophys. Res. Commun..

[40] Clarke L., Carbon J. (1976). A colony bank containing synthetic Col El hybrid plasmids representative of the entire *E. coli* genome. Cell.

[41] Marco M.L., Bongers R.S., de Vos W.M., Kleerebezem M. (2007). Spatial and temporal expression of *Lactobacillus plantarum* genes in the gastrointestinal tracts of mice. Appl. Environ. Microbiol..

[42] Bottacini F., Zomer A., Milani C., Ferrario C., Lugli G.A., Egan M., Ventura M., van Sinderen D. (2017). Global transcriptional landscape and promoter mapping of the gut commensal *Bifidobacterium breve* UCC2003. BMC Genom..

