# Supplementary material for: Application of Recombinase-Based In Vivo Expression Technology to Bifidobacterium longum subsp. longum for Identification of Genes Induced in the Gastrointestinal Tract of Mice
Source: Microorganisms. 2020 Mar 13;8(3):410. doi: 10.3390/microorganisms8030410 (PMC7143038; doi:10.3390/microorganisms8030410)
Supplement: Supplementary file 1 [file microorganisms-08-00410-s001.pdf]

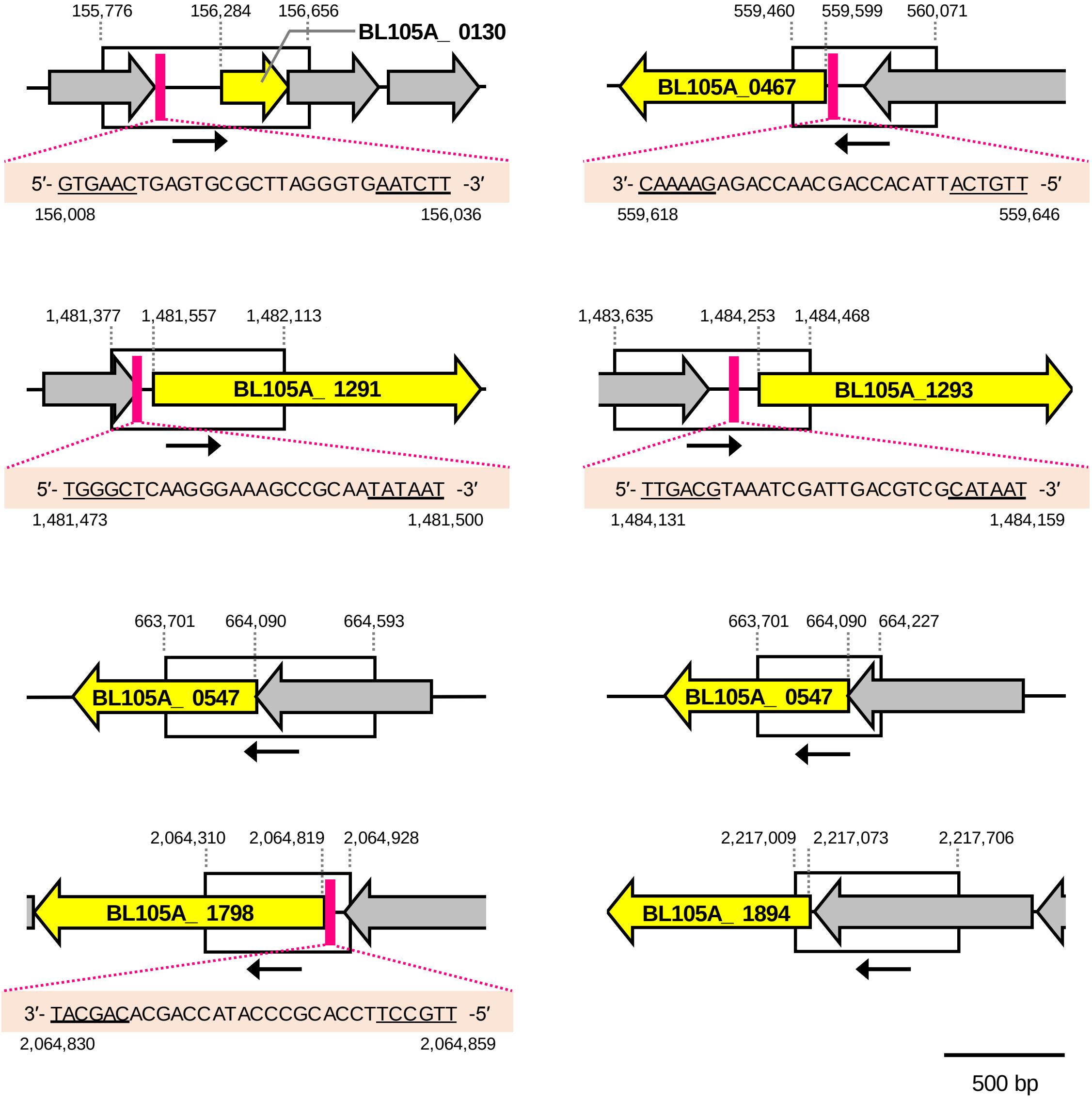

Figure S1. The Structure and genomic position of the inserted DNA fragment in representative R-IVET clones. Clones correspond to the genes used in the verification of *in vivo*-induced expression by qRT-PCR. Open boxes with a black arrow indicate the locations and orientation of the DNA fragments inserted upstream of the promoterless Cre gene. Pink boxes show the predicted promoter regions. No predictable promoter region was detected in the corresponding clones of the genes BL105A_0547 and BL105A_1894. The promoter sequences are shown in an enlarged view on the bottom of the gene structure. Normal- and bold-single underlined sequences indicate the −35 and the −10 regions, respectively. Genes used for the qRT-PCR analysis are shown by yellow arrows with a locus tag number, while the other genes are indicated by gray arrows. Genomic positions are determined based on the complete genome sequence of *B. longum* 105-A (GenBank accession no. AP014658.1). The upstream region of BL105A_0547 was identified in two distinct clones by the R-IVET (third and fourth trials, see Table 3).

Table S1. Diet compositions for the third and fourth R-IVET experiments

**Component (g/kg diet)**	**AIN-93G control diet**	**1-Kestose-containing diet**
Maltodextrin [1]	529.5	469.5
Casein [2]	200	200
Sucrose [3]	100	100
Soybean oil [4]	70	70
Cellulose [5]	50	50
Mineral mixture [6]	35	35
Vitamin mixture [7]	10	10
L-Cystine [8]	3	3
Choline chloride [9]	2.5	2.5
1-Kestose [10]		60

[1] TK-16 (Matsutani Chemical Industry Co., Ltd., Hyogo, Japan).

[2] ALACID (New Zealand Dairy Board, Wellington, New Zealand).

[3] Nippon Beet Sugar Mfg. Co., Ltd., Obihiro, Japan.

[4, 8, 9] Wako Pure Chemical Industries, Ltd., Osaka, Japan.

[5] Cellulose powder type D (Advantec Toyo Co., Ltd., Tokyo, Japan).

[6] AIN-93G Mineral mixture (MP Biomedicals. Inc., CA, USA).

[7] AIN-93G Vitamin mixture (CLEA Japan. Inc., Tokyo, Japan).

[10] B Food Science Co., LTD., Tokyo, Japan
